# Utilization of long-lasting insecticide-treated net and its associated factors among pregnant women in Dawo district, Southwest Shoa Zone, Oromia, Ethiopia, 2023

**DOI:** 10.3389/fpubh.2023.1261254

**Published:** 2024-01-29

**Authors:** Kumsa Negasa, Tufa Kolola Huluka, Mecha Aboma Yebassa, Tolossa Waqkene

**Affiliations:** ^1^Department of Public Health, Dawo District Health Office, Woliso, Ethiopia; ^2^Department of Public Health, College of Medicine and Health Sciences, Ambo University, Ambo, Ethiopia

**Keywords:** long lasting insecticide treated net (LLIN), LLIN utilization, pregnant women, malarious sub-districts, malaria, Ethiopia

## Abstract

**Introduction:**

The use of long-lasting insecticide-treated net (LLIN) is one of the most effective malaria prevention strategies, particularly among pregnant women. It has mosquito-killing repellent and physical barrier properties. Pregnant women, children under 5 years of age, and patients with HIV/AIDS, as well as non-immune migrants, mobile populations, and travelers, are the most vulnerable groups to malaria. Even when pregnant women are given priority, not all nets owned by households are used by pregnant women. Therefore, the risk of the occurrence of malaria during pregnancy is also high.

**Objective:**

This study aimed to assess the utilization of long-lasting insecticide-treated net and its associated factors among pregnant women in Dawo district, Southwest Shoa Zone, Oromia, Ethiopia, 2023.

**Methods:**

A community-based analytical cross-sectional study was carried out in Dawo district, Southwest Shoa Zone, Oromia, Ethiopia, from 21 to 30 April 2023. A total of 353 pregnant women were chosen through simple random sampling and interviewed face-to-face using a structured and pre-tested questionnaire. Before being exported to the Statistical Package for Social Science (SPSS) version 23, the data were coded and entered into EPIDATA version 4.6. The study examined the prevalence of long-lasting insecticide-treated net use among pregnant women in the Dawo district using descriptive statistics. Analytical statistics, such as bivariable and multivariable logistic regression analyses, were used to determine the relationship between independent and dependent variables. Statistical significance was determined using a *P*-value of <0.05 and adjusted odd ratios with 95% confidence intervals.

**Results:**

Long-lasting insecticide-treated nets were utilized by 55.5% (95% CI*:* 50.4–60.7%) of all pregnant participants in the study, which was below the national target. Pregnant women who have antenatal care (ANC) contact for current pregnancy adjusted odds ratio (AOR = 4.66, 95% CI: 1.95, 11.10), community-based health insurance (CBHI) enrollment (AOR = 2.38, 95% confidence intervals, CI: 1.38, 4.11), children under 5 years of age (AOR = 2.68, 95% CI: 1.62, 4.43), understanding that malaria poses a risk to fetuses (AOR = 3.25, 95% CI: 1.26, 8.41), and LLINs access (AOR = 12.47, 95% CI: 3.98, 39.08), were factors that significantly associated with LLIN utilization.

**Conclusion:**

In conclusion, the utilization of LLINs was relatively low. ANC contact for current pregnancy, CBHI enrollment, having children under the age of 5, having a high income, and understanding that malaria poses a risk to fetuses were factors significantly associated with LLIN utilization among pregnant women.

## 1 Introduction

The use of long-lasting insecticide-treated net (LLIN) is one of the most effective strategies for the prevention of malaria, especially among pregnant women. LLINs perform three primary functions: (1) Mosquitoes are knocked down, temporarily incapacitated, or even killed when exposed to the net; (2) They act as a repellent; (3) They reduce contact between the person sleeping under the net and mosquitoes by acting as a physical barrier (1). When used in good condition, LLINs were also associated with a decreased risk of the occurrence of malaria during pregnancy (2).

The World Health Organization (3) and the President's Malaria Initiative—the Ethiopian Malaria Operational Plan/2020 recommend 100% coverage and 80% LLIN usage by high-risk groups. It aimed to achieve universal 100% coverage in malaria-endemic areas with pregnant women owning at least one LLIN per sleeping space or per two people on average and by achieving and maintaining utilization above 80% by all age and sex groups (conditional upon LLINs remaining effective). The WHO encourages the continued use of the nets to prevent malaria (3–5).

LLINs protect pregnant women from malaria, lowering the risk of anemia, abortion, stillbirth, and maternal death. LLINs also help newborns by lowering the incidence of low birth weight, lowering the incidence of newborn anemia, lowering the risk of newborn death, and promoting growth and development during pregnancy and the first few weeks of life (1, 4).

Over the last two decades, there has been an increase in access to malaria prevention methods and tools, such as LLINs. Ethiopia distributed ~80 million LLINs between 2006 and 2016, with the goal of providing universal coverage of one free LLIN for every two people in high-risk areas (6).

However, LLIN use is quite low among pregnant women in Sub-Saharan Africa (SSA), and the percentage of households in Ethiopia that own and utilize LLINs continues to be much below national and WHO recommendations, with reported variables including residence, age, family size, income, education, attitude, and other considerations (6–9).

In a district-wide campaign, LLINs were supplied to sub-districts at risk for malaria. If any LLINs were damaged or lost, replacements could be made by health extension workers during house-to-house visits. It is also delivered in conjunction with other public health efforts that target young children and pregnant women, such as the expanded immunization program and ANC services, which reach a sizeable number of the target populations and offer free services. For 12 years, or three or four distribution cycles, only long-lasting insecticidal nets (LLINs) were supplied throughout the district.

Furthermore, no study on LLIN utilization among pregnant women in the Dawo district has been conducted that takes into account LLIN utilization at the household level and associated factors. The goal of this study was to close this gap by looking at LLIN use and its associated factors among pregnant women in the Dawo district of Southwest Shoa, Ethiopia.

## 2 Methods and materials

### 2.1 Study area and period

Dawo district is one of the administrative districts of Oromia region's Southwest Shoa zone, located between 36 00′ 00′′ and 39 00′ 00′′ N and 39 00′ 00′′ and 42 20′ 00′′ E and ~96 km west of Addis Ababa, the capital of Ethiopia. It has 23 rural sub-districts, the smallest administrative unit. The district also has four health centers and 23 health posts, each of which has 100% health coverage. According to the 2023 calendar, there were 108,702 people in the district, with 54,384 males and 54,318 females. In the district, there were an estimated 24,056 reproductive females and 3,772 pregnant females.

**Figure 1:**
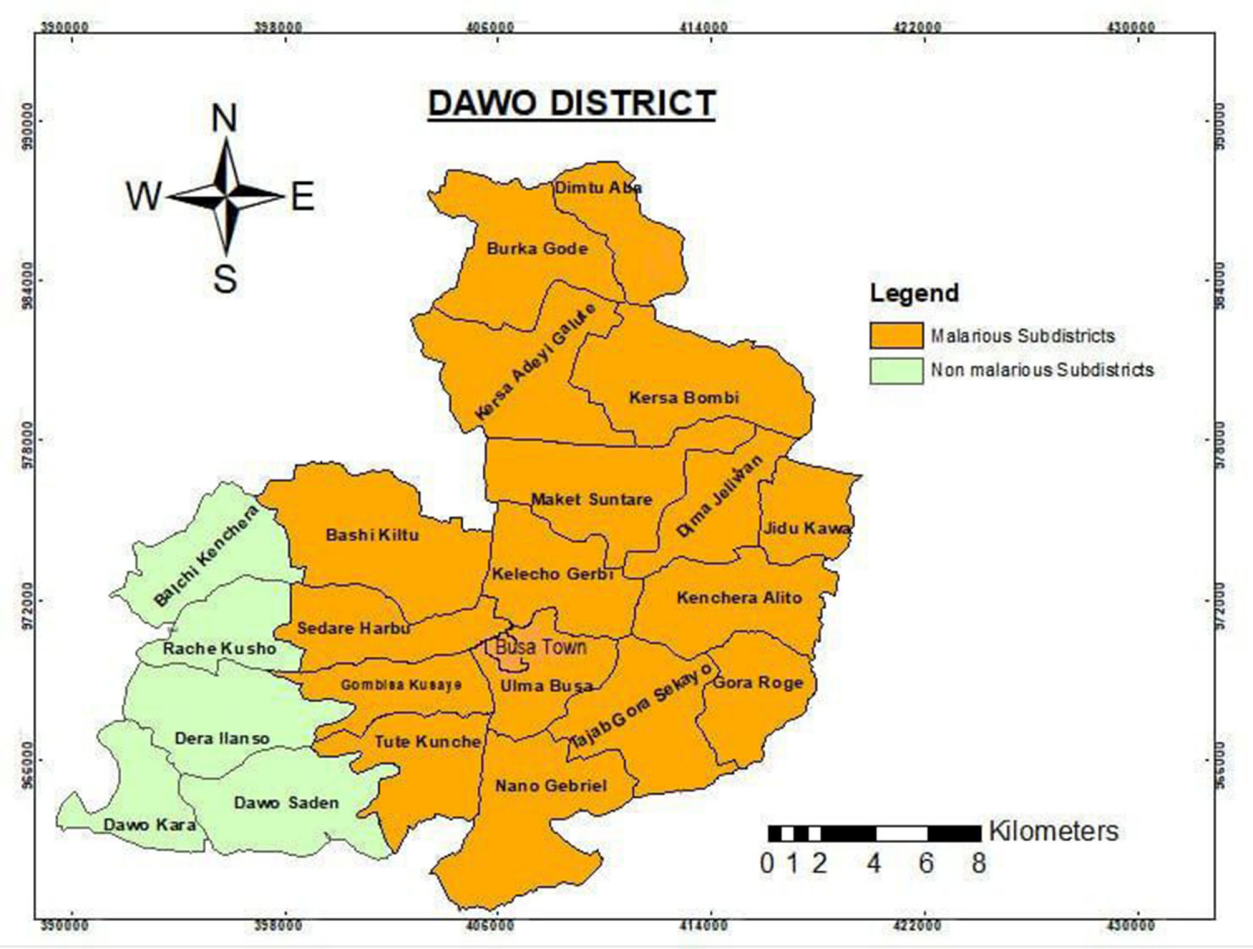
Administrative map of Dawo district.

The weather in the district is mostly “Badda-Daree” (hot), especially during the study period, and ~18 sub-districts (78%) and 89,954 (83%) populations are at risk of contracting the infectious disease malaria. Given that pregnant women are a vulnerable group to malaria infection, they were expected to utilize the LLINs constantly, and the operational strategy for its elimination also encouraged studies that backed it up. As a result, this district was carefully chosen for this study since it is a malaria hotspot area and there has not been any other comparable research there. The research was conducted from 21 to 30 April 2023 (Figure 1).

### 2.2 Study design

A community-based analytical cross-sectional study was conducted in the Dawo district.

### 2.3 Population

#### 2.3.1 Source population

The source populations were all pregnant women living in the Dawo district.

#### 2.3.2 Study population

All randomly selected pregnant women eligible for the study were included. Eligible pregnant women were those who spent the night at home before the interviewer's visit.

#### 2.3.3 Study unit

Pregnant women from whom actually the data were collected.

#### 2.3.4 Inclusion criteria

Pregnant women who lived in selected malarious sub-districts of Dawo district for 6 months or more prior to the data collection period and were registered by health extension workers (HEWs) of the study area for the identification of pregnant women for ANC follow-up and other obstetric services, or a woman who self-reported herself as pregnant during the study period, were included in the study.

#### 2.3.5 Exclusion criteria

Pregnant women who were critically ill and unable to respond during data collection were assumed to be excluded, but there were no refusals in this study.

### 2.4 Sample size determination and sampling techniques

#### 2.4.1 Sample size determination

The required sample size for the primary objective was determined using Epi-version 7 software based on the following assumptions: single population proportion for a cross-sectional survey, assuming a proportion (*p*) of 52.5% (10) of LLINs used among pregnant women based on a previous study, and assuming a 5% margin of error (*d* = 0.05) at the 95% confidence level and a 10% adjustment for non-response.

n = (Zα/2)^2^ p^*^(1 – p)/d^2^

n = (1.96)^2^ 0.525^*^0.475/(0.05)^2^

*n* = 383, since the target population was <10,000 as estimated by the district annual plan, the limited population revision factor was applied to determine the final sample size.

nf = Nn/N+n−1 (11).

where *n* = calculated sample size before adjustment.

*N* = target population size (pregnant women in malarious sub-districts) of 1,810.

nc = sample size after a finite population correction factor.

nc = 1,810^*^383/1,810 + 383 – 1

nc = 693,230/2,192

nc = 317

nf = nc/1-λ, where λ is an adjustment for non-response rate (10%)

nf = 317/0.9

nf = 353, (including a 10% non-response rate).

The sample size for the second objective was determined using variables from the same study. ANC follow-up and family size were taken, and the sample size was calculated with a level of confidence of 95% and a power of 80%, yielding 216 (OR = 2.39) and 48 (OR = 6.64,) samples, respectively. Since the first sample was the largest of the three, it was the final sample, which was 353.

#### 2.4.2 Sampling techniques and procedures

**Figure 2:**
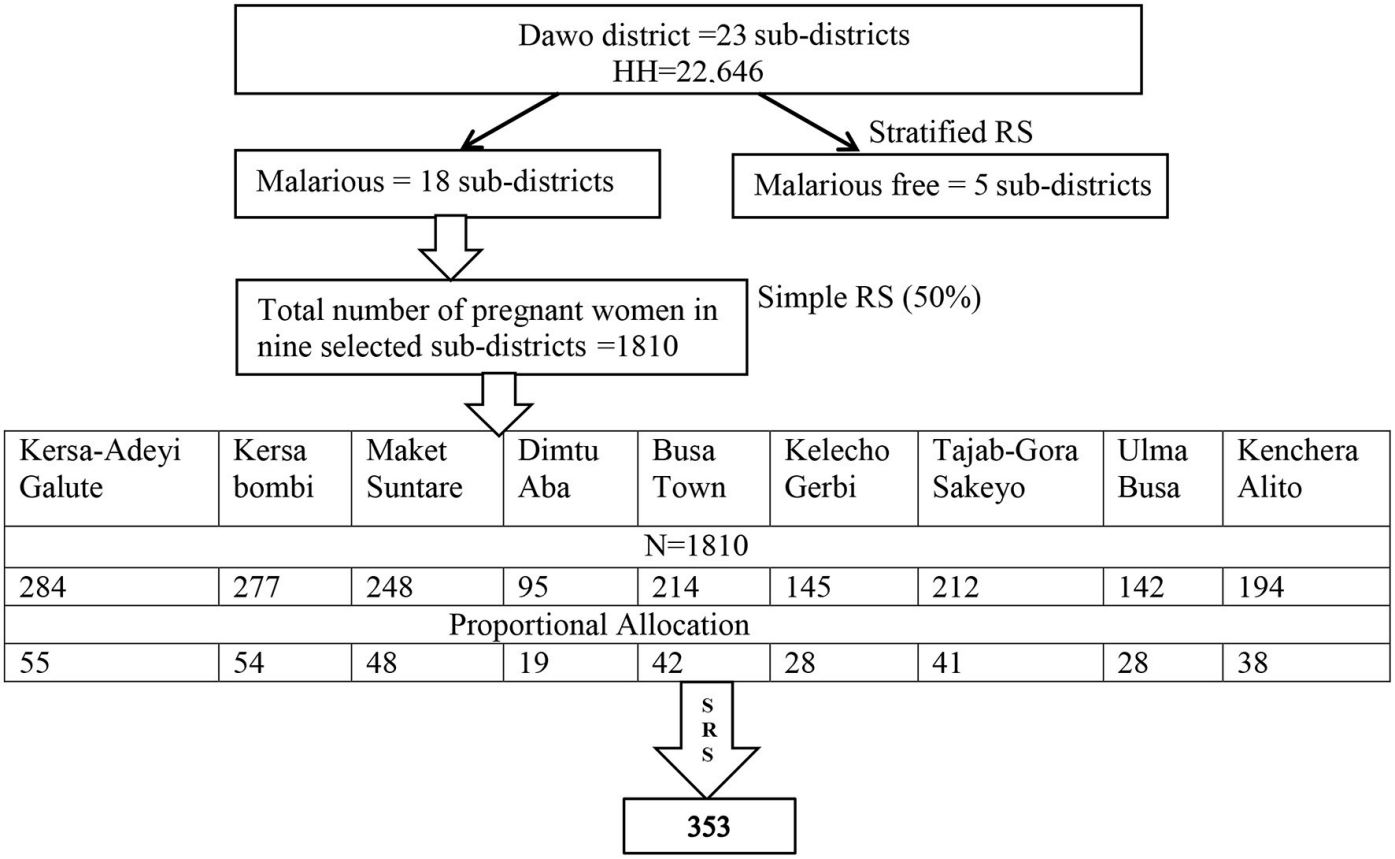
Schematic presentation of a sampling procedure for the utilization of LLINs and its associated factors among pregnant women in the Dawo district of Southwest Shoa, Oromia, Ethiopia, 2023 (12).

The simple random sampling method was used to choose 9 (50%) of the district's 18 malarious sub-districts that provide a representative sample of study subjects. The total number of households with pregnant women was obtained from the district's annual pregnant women estimation and ANC registration at selected health centers and health posts. Health extension workers in each sub-district were trained on how to identify pregnant women based on the self-report of the woman during the study period or the head of the household as the women were pregnant in order to draw a sampling frame from the selected sub-district. The sample size was assigned to each selected sub-districts using proportional allocation. The study included households with pregnant women from the selected sub-districts using simple random sampling (Figure 2).

### 2.5 Variables

#### 2.5.1 Dependent variable

LLIN utilization

#### 2.5.2 Independent variables

- ✠ **Sociodemographic factors**

LLITN use was influenced by age, CBHI enrollment status, education, occupation, income, and marital status.

- ✠ **LLINs provision, ownership, and information access**

LLIN utilization might be affected by the frequency of distribution, LLIN access, information access, LLIN use perception, knowledge of malaria disease, and consistency of LLIN use.

- ✠ **Household characteristics**

Family size, number of under-5-year-old children in the house, housing type, number of sleeping rooms, and area of residence were important factors.

- ✠ **Obstetric factors and ANC follow-up**.

Parity, stage of current pregnancy, and number of antenatal care contacts for current pregnancy might influence utilization.

### 2.6 Operational definition

**LLINs** are a factory-treated net that does not require any treatment. It is designed to maintain efficacy against mosquito vectors for at least 3 years.

**LLIN ownership** was households having one or more LLINs during the home visit.

**LLIN utilization** was a mother who was reported to have slept under LLIN during the night prior to the survey date.

**Pregnant woman** was a human female who was registered for ANC follow-up by HEWs or a woman who self-reported herself as pregnant during the study period.

### 2.7 Data collection tools and techniques

In accordance with the Roll Back Malaria recommendations, a semi-structured questionnaire and observational checklists were used to assess LLIN use among pregnant women. These tools were developed from the 2013 malaria indicator study (13). Its goal was to gather data from participants on sociodemographic traits, obstetric variables, and awareness of and use of LLINs by data collectors who interviewed all pregnant women at home. The information was gathered by going from house to house, and the purpose of this was to validate self-reported information by direct observation. Each expectant woman was watched after the interview to verify that using LLIN the night before was indeed done in that way. During the data collection, both observational and self-reported data for LLIN utilization were gathered. To ensure consistency in meaning and rationality, the questionnaire was developed in English first, then translated into Afan Oromo, and then back-translated into English by language experts. Six trained nurses as data collectors and two health officers as supervisors collected data for LLIN utilization using interviewer-administered questionnaires and direct observation of LLIN usage. Pregnant women's reports of using LLINs were supplemented by the observation of the pregnant women's households as they hung (mounted) LLINs over their beds or sleeping areas. Pregnant women sleeping under LLINs during the early morning hours of the observation day were classified as using them, and vice versa.

### 2.8 Data quality control and management

An appropriate data collection instrument was tailored to ensure data quality. A pretest was performed on 5% of the sample size in a nearby district, Bacho. This was not one of the main study's recruitment sites. To ensure consistency, the questionnaire was written in English and translated into Afan Oromo before being back-translated to English by language experts. Six nurses from public health centers with prior data collection experience and fluency in the local language were hired for data collection. Two public health officers were assigned as supervisors and supervised the data collectors for proper data collection. Data collectors and supervisors received 2 days of training. Training was provided on the study's objectives, data collection methods, questionnaire completeness, and how to approach households. The investigator and supervisor checked and reviewed questionnaires on a daily basis to ensure that the information collected was complete and consistent. On a daily basis, feedback on previous data activities was provided to both data collectors and supervisors.

### 2.9 Data processing and analysis

The collected data were coded and entered into Epi-Data version 4.6 for cleaning before being transferred to SPSS version 23.0 for analysis. To summarize the data, descriptive statistics such as frequencies and percentages were used. Descriptive analysis (frequency and percentages) was utilized to ascertain sociodemographics, obstetrics, knowledge of LLINs, and the prevalence of LLIN utilization among pregnant women. The skewness and kurtosis *z*-values, Shapiro–Wilk test *p*-value, histogram, normal Q–Q plots, and box plots were used to examine the normality of the data distribution. Bivariable and multivariable analyses were used to identify factors associated with LLIN utilization among pregnant women. The multi-variable logistic regression model was used following the design phase's restriction of LLIN utilization and its associated factors among pregnant women, which helped to adjust for confounding variables. Variables with a *p*-value of 0.25 that show an association in the bi-variable analysis were entered into a multivariable logistic regression model. Hosmer–Lemshow's goodness of fit test was used to determine whether the required assumptions for the application of multi-variable logistic regression were met. Standard error was used to assess multicollinearity, and the variables were entered into the multi-variable model without multicollinearity. Adjusted odds ratios (AORs) with 95% CIs were computed, and statistical significance was declared at a *p*-value of 0.05.

### 2.10 Ethical and legal considerations

The Institutional Review Board (IRB) of Ambo University's College of Medicine and Health Sciences provided ethical approval under the reference number AU/PGC/589/2015 prior to data collection. The Southwest Shoa Zone Health Department and the Dawo District Health Office were also contacted for written permission. Prior to data collection, each study participant was provided with informed verbal consent. The study's goal, risks, and benefits were all communicated in the local language, and participants were given adequate time to ask questions/clarify their understanding. The participants were also guaranteed the privacy and confidentiality of the data obtained. Pregnant women were allowed to refuse to participate or withdraw from the research without regard for the services provided by healthcare institutions.

## 3 Results

### 3.1 Sociology-demographic characteristics of pregnant women

In this research study, a total of 353 pregnant women were interviewed, with a 100% response rate. Their mean age (±SD) was 25.04 (±5.47) years, and the class mode of their age was between 15 and 24 years with 48.7% of the respondents. Over 90% of the respondents were married, and regarding their level of education, approximately three-fourths (73.4%) had formal education. The respondents were asked about their employment status, and the majority (72.5%) were farmers (Table 1).

**Table 1:** Sociodemographic characteristics of pregnant women in Dawo district, South West Shoa, Oromia, Ethiopia, 2023.

**Variables**	**Category**	**Frequency (*n* = 353)**	**Percent (%)**
Age in year	15–24	172	48.7
	25–34	152	43.1
	35–44	29	8.2
Marital status	Married	326	92.4
	Single	10	2.8
	Others^**^	17	4.8
Residence	Rural	311	88.1
	Urban	42	11.9
Women's education	No formal education	94	26.6
	Primary education	169	48
	Secondary education	71	20
	College and above	19	5.4
Women's occupation	Farmer	256	72.5
	Housewife	40	11.3
	Merchant	28	7.9
	Government employee	17	4.8
	Daily laborer	12	3.4
Family size	≤ 4	243	68.8
	>4	110	31.2
Average monthly income	< 55 USD	187	53
	≥55 USD	166	47
Religion	Orthodox	255	72.2
	Protestant	93	26.3
	Other^*^	5	1.4
CBHI membership status	Yes	251	71.1
	No	102	28.9

^**^Divorced, separated, and widowed.

^*^Muslims.

### 3.2 Obstetrics characteristics of pregnant women

In terms of the respondents' parity, the majority (80.5%) were multipara. The second trimester, which accounts for 41.9% of all pregnancies among respondents, is followed by the third trimester, which accounts for 40.5%. Nine out of 10 (89.9%) pregnant women were in contact with ANC during their pregnancy, and at least 30.6% of them have had two visits (Table 2).

**Table 2:** Obstetrics characteristics of pregnant women in Dawo district, South West Shoa Oromia, Ethiopia, 2023.

**Variables**	**Category**	**Frequency (*n* = 353)**	**Percent (%)**
Parity	Primipara	69	19.5
	Multipara	284	80.5
Stage of current pregnancy	First trimester	62	17.6
	Second trimester	148	41.9
	Third trimester	143	40.5
Attend ANC	Yes	317	89.8
	No	36	10.2
Number of ANC contacts (317)	First contact	74	23.3
	Second contact	97	30.6
	Third contact	91	25.7
	Fourth contact and above	55	17.4

### 3.3 Knowledge of LLINs among pregnant women

The majority (91.8%) of the pregnant women who responded have heard about LLINs, and more than half (66.7%) of them heard about them through healthcare professionals. According to the comments of the respondents, under-5-year-old children (20.7%) and pregnant women (54.6%) were the groups with the highest risk of contracting malaria. LLINs kill mosquitoes, which was the response of more than half (54.7%) (Table 3).

**Table 3:** Knowledge of LLINs among pregnant women in Dawo district, South West Shoa, Oromia, Ethiopia, 2023.

**Variables**	**Category**	**Frequency (*n* = 353)**	**Percent (%)**
Heard about LLINs	Yes	324	91.8
	No	29	8.2
Source of information (324)	Health worker	216	66.7
	TV	55	17
	Radio	53	16.3
knowledge of malaria high-risk groups	Pregnant women	177	54.6
	Under five children	67	20.7
	Older adults	26	8.0
	Equally serious for all	46	14.2
	Others	8	2.5
Knowledge of malaria prevention methods or ways of protection	Knows at least one method	39	11
	Knows ≥2 methods	310	87.8
	Other^*^	4	1.2
Knowledge of benefits of LLINs	Protects from bite of mosquitoes or physical barrier	89	25.2
	Kill mosquitoes	193	54.7
	Protects from malaria	65	18.4
	Others^**^	6	1.7

^**^Used as a curtain, as mattress.

^*^Don't know.

### 3.4 Ownership and utilization of LLINs among pregnant women

There were 658 LLINs recorded among the 1,263 residents of the examined houses (*n* = 328). The total average of LLIN ownership per household was 2.00 (658/328), with 1.92 (1,263/658) people per LLIN (computed in homes that possessed nets). Approximately 93% (92.9%) of homes owned at least one LLIN. Generally, there was a wide gap between ownership and utilization of LLINs.

Observational utilization of LLINs by pregnant participants in the study was 55.5% (95% CI: 50.4–60.7%), which was lower than the national target. Concerning the consistency of utilization of LLINs, nearly half (49%) of the users were utilizing LLINs throughout the night during the season for mosquitoes (Table 4).

**Table 4:** Ownership and utilization of LLINs among pregnant women in Dawo district, South West Shoa, Oromia, Ethiopia, 2023.

**Variables**	**Category**	**Frequency (*n* = 353)**	**Percent (%)**
Owns LLINs	No	25	7.1
	Yes	328	92.9
Utilize LLINs	No	157	44.5
	Yes	196	55.5
Frequency of LLIN utilization (*n* = 196)	Throughout the night during the season for mosquitoes	96	49.0
	All year round	80	40.8
	Most part of the night	17	8.7
	Other^*^	3	1.5

^*^Some part of the night.

### 3.5 Reasons for not sleeping under LLINs

**Figure 3:**
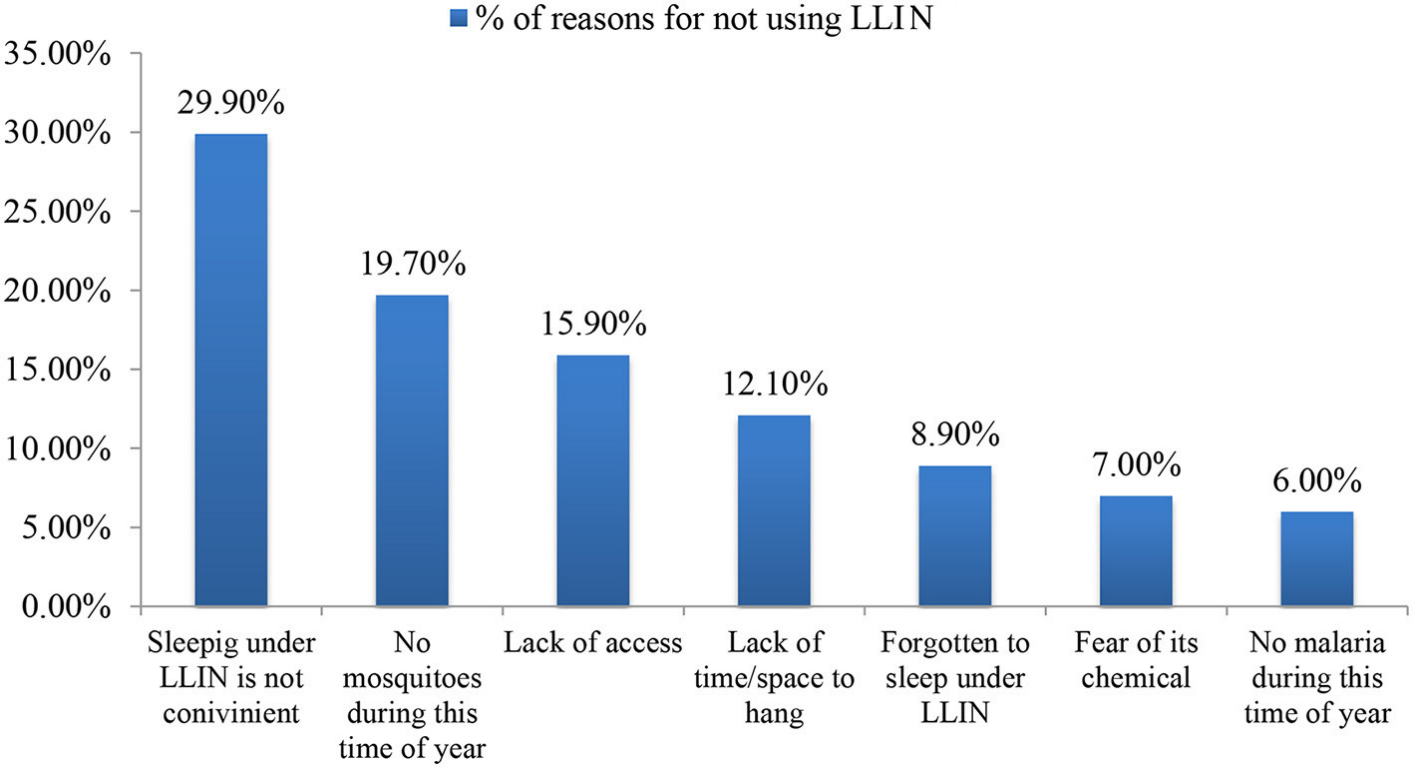
Reasons for not sleeping under LLINs (*n* = 157).

When asked why they did not use LLINs, the majority (29.9%) reported that they were uncomfortable or inconvenient, followed by the assertion that there were no mosquitoes at this time of year (19.7%) and that LLINs were not readily available (15.9%) (Figure 3).

### 3.6 Factors associated with LLIN utilization among pregnant women

In the bi-variable analysis, gestational age, number of ANC contacts for the current pregnancy, parity, having children under the age of 5 in the home, CBHI enrollment status, awareness or having information on LLINs use, average monthly income, access to LLINs within the last 12 months, and knowledge about the risk of malaria to the fetuses were factors significantly associated with LLIN utilization.

In the multivariable logistic regression, pregnant women who have ANC contact for their current pregnancy had 4.66 times more likelihood of using LLINs compared to those who had no ANC contact (AOR = 4.66, 95% CI: 1.95, 11.10).

Pregnant women who were CBHI enrolled had a 2.38 times higher chance of using LLINs than pregnant women who were not (AOR = 2.38, 95% CI: 1.38, 4.11). Similar to this, pregnant women who have under-5-year-old children were 2.68 times more likely to use LLINs than pregnant women who do not have any children under the age of 5 (AOR = 2.68, 95% CI: 1.62, 4.43).

Additionally, compared to their counterparts, pregnant women who understood that malaria poses a risk to fetuses were 3.25 times more likely to use LLINs (AOR = 3.25, 95% CI: 1.26, 8.41). There was a statistically significant association between access and LLIN use (AOR = 12.47, 95% CI: 3.98, 39.08). Those who accessed LLIN within the last 12 months were 12.47 times more likely to utilize it than those who did not (Table 5).

**Table 5:** Bivariable and multivariable logistic regression analyses for factors associated with LLIN utilization among pregnant women in Dawo district, South West Shoa, Oromia, Ethiopia, 2023.

**Variables**	**Category**	**Utilize LLIN**		**COR (95% CI %)**	***P*-value**	**AOR (95% CI %)**	***P*-value**
		**Yes (*n*)**	**No (n)**				
CBHI membership status	Yes	155	96	2.40 (1.50, 3.85)	0.002^**^	2.38 (1.38, 4.11)	0.002^**^
	No	41	61	1.00		1.00	
Average monthly income	< 55 USD	91	96	1.00	0.006^**^	1.00	0.002^**^
	≥55 USD	105	61	1.82 (1.19, 2.78)		2.20 (1.34, 3.62)	
Having under 5 years children	Yes	103	53	2.17 (1.41, 3.35)	< 0.001^**^	2.68 (1.62, 4.43)	≤ 0.001^**^
	No	93	104	1.00		1.00	
ANC contact for current pregnancy	Yes	187	130	0.23 (0.11, 0.51)	< 0.001^**^	4.66 (1.95, 11.10)	≤ 0.001^**^
	No	9	27	1.00		1.00	
Access to LLINs use information	Yes	191	133	6.89 (2.56, 18.53)	< 0.001^**^	4.40 (1.42, 13.66)	0.010^*^
	No	5	24	1.00		1.00	
Awareness of malaria as a communal issue	Yes	180	122	3.44 (1.80, 6.58)	< 0.001^**^	1.00	
	No	15	35	1.00			
Understanding that malaria poses a risk to fetuses	Yes	187	134	3.57 (1.60, 7.95)	0.002^**^	3.25 (1.26, 8.41)	0.015^*^
	No	9	23	1.00		1.00	
LLINs access within the last 12 months	Yes	192	131	9.53 (3.25, 27.94)	< 0.001^**^	12.47 (3.98, 39.08)	≤ 0.001^**^
	No	4	26	1.00		1.00	

^*^Statistically significant at a *p*-value of < 0.05.

^**^Statistically significant at a *p*-value of < 0.01.

The qualitative portion of the study, which was based on open-ended survey questions and in-depth interviews, further confirmed that hanging a rectangular net in a small multipurpose room is uncomfortable and challenging to maintain in the hung position for an extended period of time. The qualitative open-ended questions and the observations revealed that numerous LLINs were mishandled and in poor condition. Some households have used LLINs to cover or wrap crops and dairy goods from the field or to utilize the nets for reasons other than those for which they were designed, which might reduce the LLINs' effectiveness and worsen the condition of the nets. The perception that LLINs are poor at eliminating bedbugs and other arthropods has been identified as a significant obstacle to continued usage of the nets, undercutting the severity of the malaria problem. The effectiveness of LLINs that do not provide flea and bedbug protection after the first few months of usage was questioned by some interviewees.

## 4 Discussion

The objective of this study was to assess the utilization of LLINs and its associated factors among pregnant women in the Dawo district. As a result, this study offers crucial information on the factors that influence LLIN use among pregnant women. The observational utilization of LLINs by pregnant participants in the study was 55.5% (95% CI: 50.4–60.7%), which was lower than the national target. This result is consistent with research from Limmu-Seka (52.5%) (10), East Bellessa (56.5%) (14), Sodo Zuria (56.9%) (15), and multi-modeling of individual, community, and regional factors associated with LLINs usage among pregnant women (58.37%) (16) studies.

Despite the fact that malaria is widespread in this study location, fewer pregnant women used LLINs than were found in Ghana (94.8%) (17), Rwanda (87.6%) (18), South Eastern Nigeria (70%) (19), Zambia (68%) (20), Damot district (72.5%) (21), and Fogera district (68.3%) (22). The varied geographic locations, socioeconomic situations, and approaches to malaria control in the aforementioned nations may be the reason for these differences. Additionally, this is because this study was conducted in a stable transmission area, and the majority of the pregnant women did not attend or lately attended ANC contact for the first time, which might increase their missed opportunity for access to LLINs and malaria prevention information. Currently, the country's main priorities are to level down the number of malaria cases across all stages and areas, and due to variances in the frequency of mosquito bites and malaria infections as well as access to health information, special attention and resource allocations must be required in terms of strategy and activities to control malaria in these areas.

Several sociodemographics, obstetrics, knowledge of malaria disease, and LLIN-related factors were entered into backward regression to determine what factors influence LLIN utilization, and the results revealed a significant association between average monthly income, CBHI enrollment status, having under-5-year-old children, understanding that malaria poses a risk to fetuses, having ANC contact for current pregnancy, LLINs access within the last 12 months, and having information on LLIN utilization.

Comparatively to their counterparts, pregnant women with average monthly incomes of more than or equal to 55 USD had a 1-fold increased odds of using LLINs. Pregnant women with high average monthly incomes had a higher likelihood of using LLINs, according to studies from SSA and northwestern Ethiopia (22–24). This study indicates that financially empowering women may help promote their health and general wellbeing.

Women who had ANC contact during their current pregnancy had 4.66 times greater odds of utilizing LLINs than women who did not have ANC contact during their present pregnancy. Pregnant women who attended ANC at least once had a 1.72 times higher likelihood of using LLINs. Women who are further along in their ANC contacts are more likely to be informed by health professionals on malaria prevention measures and utilize LLINs. Similarly, the studies covering Awabel, Sodo Zuria, and Miesso districts noted that women with ANC were more likely to use LLINs (6, 9, 15). There is evidence to support the idea that physicians' counseling during ANC is essential for increasing the use of LLINs.

Furthermore, this study identified factors associated with LLIN utilization among pregnant women and discovered that being a member of CBHI was a factor that was significantly associated with LLIN utilization. Comparatively speaking to CBHI enrollment, the odds of using LLINs for pregnant women who had access to CBHI were 2.38 times higher than those who did not have access to it. The reason could be explained by mothers from CBHI households who may be exposed to health institutions for various health service utilizations and thus may have received information or awareness about LLINs. This study found that age, marital status, occupation, number of sleeping rooms, family size, and location of residence had no effect on expectant women's use of LLINs. This is supported by research conducted in northern Ethiopia (25). However, the studies in northern Ethiopia and Rwanda reported that old-aged women and married were more likely to utilize LLINs compared to their counterparts, respectively (11, 18, 24).

Pregnant women who recognize that malaria poses a risk to their fetuses are 3.25 times more likely to use LLINs than their counterparts. This theory is confirmed by a study conducted in Pakistan, which found that women utilize LLINs more if their prior pregnancy resulted in a neonatal death (26).

The likelihood of LLIN use was 4.4 times higher among pregnant women who had LLIN use information than among pregnant women who did not have information. This idea is also evidenced by the study conducted on the effect of malaria preventive education on the use of LLINs among pregnant women in Nigeria (27).

The odds of utilizing LLINs by pregnant women with children under five were 2.68 times greater than that of their contemporaries. A study conducted in Uganda that showed households used LLINs more when there were children under 5 in the home further confirmed this (23). The main limitation of LLIN utilization in this study was a lack of LLINs in households. When compared to women who had no access to LLINs in the previous year, those who had access were 12.47 times more likely to use LLINs This hypothesis is reinforced by a study conducted in southwest Ethiopia, which found that households use LLINs when they have sufficient access (23, 28).

The qualitative portion of the study, which was based on open-ended survey questions and in-depth interviews, further confirmed that hanging a rectangular net in a small multipurpose room is uncomfortable and challenging to maintain in the hung position for an extended period of time. The qualitative open-ended questions and the observations revealed that numerous LLINs were mishandled and in poor condition. Some households have used LLINs to cover or wrap crops and dairy goods from the field or to utilize the nets for reasons other than those for which they were designed, which might reduce the LLINs' effectiveness and worsen the condition of the nets. The perception that LLINs are poor at eliminating bedbugs and other arthropods has been identified as a significant obstacle to continued usage of the nets, undercutting the severity of the malaria problem. The effectiveness of LLINs that do not provide flea and bedbug protection after the first few months of usage was questioned by some interviewees.

### 4.1 Strengths and limitations

The study's strength lies in that it was supplemented with observation to assess LLIN utilization and its associated factors among pregnant women in the district. The study was also carried out in an area where malaria was important for public health, and better scheduling was employed to evaluate utilization within a fiscal year after long-lasting net distribution to the community.

The analytical cross-sectional study design that was used could not establish a temporal relationship (cause and effect) between the outcomes and response variables since the data were collected at a single point in time. The community has a tendency to highlight socially acceptable conduct and downplay socially stigmatized behavior; therefore, there may be a social desirability bias present when LLIN usage is observed. In order to reduce social desirability bias, the interview questions were carefully constructed with a hint of sensitivity and subtlety. Pregnant women were also asked questions in a circumspect manner with some finesse and empathy, and participants were assured of complete confidentiality. Anonymity was permitted, and participants were assured of complete confidentiality during the interview. Some replies may be affected by recall bias, which would also have an impact on the study's outcomes. However, in this study, the only information that participants needed to remember was whether they had slept beneath an LLIN the night before as well as the existence and quantity of LLINs in the home. In order to reduce observation mistakes, the data collectors were trained on how to get accurate information from participants and how to observe the use of LLINs at the site. Supervisors also made sure that there were no discrepancies between the information gathered and the observed utilization status and that no observation bias was present during data collection. Finally, because the design is quantitative, it does not address the respondents' cultural concerns.

## 5 Conclusion

The utilization of LLINs among pregnant women in this study was very low. Only six out of 10 pregnant women had utilized LLINs, which is lower than the target set by the Ministry of Health for LLIN coverage by 2,025 among vulnerable people. Being a member of CBHI, having a high income, stage of current pregnancy, having under-5-year-old children, understanding that malaria poses a risk to fetuses, and having access to LLINs within 12 months were factors significantly associated with LLIN utilization. The government and health sectors should educate pregnant women to engage in early ANC and CBHI and raise awareness about malaria disease to increase literacy utilization.

## 6 Recommendations

The following suggestions and future research directions are provided based on the results obtained and the difficulties encountered during the study period:

I. The government and NGOs should allocate budgets for LLIN requirements and maintain the frequency of distribution for vulnerable groups as well as every household to prevent pregnant women from contracting malaria. Additionally, the government should support or intensify economic empowerment for pregnant women, which would increase health-seeking behavior and enhance LLIN utilization.
II. Dawo District Health Office: As part of public health measures, there should be a need for effective linkages between malaria elimination and maternal health service programs in order to improve the success of efforts to control malaria during pregnancy. Therefore, the district health office should develop appropriate planning and implementation strategies that include LLIN use, advocacy and social mobilization on engagement in CBHI, awareness of malaria as a risk to fetuses, and related services that help in assessing its progress toward national targets and increasing LLIN utilization.
III. Healthcare providers should identify pregnant women as early as possible, give adequate health education to all pregnant women, and encourage early ANC contact to access and encourage consistent utilization of LLINs. Adequate health education should also be given to pregnant women on CBHI enrollment, malaria disease, and LLIN use and safety to enhance LLIN utilization among pregnant women and related interventions to prevent pregnant women from contracting malaria.
IV. Researchers should conduct research on the misuse of LLINs, the life cycle of LLINs, and the perceptions of LLINs among pregnant women, both quantitatively and qualitatively.

## Data availability statement

The original contributions presented in the study are included in the article/supplementary material, further inquiries can be directed to the corresponding author.

## Ethics statement

The studies involving humans were approved by the Institutional Review Board (IRB) of Ambo University's College of Medicine and Health Sciences provided ethical approval under the reference number of Ref. No. AU/PGC/589/2015 prior to data collection. The Southwest Shoa zone Health Department and the Dawo District Health Office were also contacted for written permission. Prior to data collection, each study participant was provided with informed verbal consent. The study's goal, risks, and benefits were all communicated in the local language, and participants were given adequate time to ask questions/clarify their understanding. The participants were also guaranteed of the privacy and confidentiality of the data obtained. Pregnant women were allowed to reject to participate or withdraw from the research without regard for the services provided by health care institutions. The studies were conducted in accordance with the local legislation and institutional requirements. Written informed consent for participation was not required from the participants or the participants' legal guardians/next of kin in accordance with the national legislation and institutional requirements.

## Author contributions

KN: Conceptualization, Formal analysis, Methodology, Software, Visualization, Writing—review & editing, Data curation, Resources, Validation, Writing—original draft. TH: Conceptualization, Formal analysis, Methodology, Software, Validation, Visualization, Writing—review & editing, Supervision. MY: Conceptualization, Formal analysis, Methodology, Software, Supervision, Validation, Visualization, Writing—review & editing. TW: Conceptualization, Formal Analysis, Methodology, Software, Visualization, Writing—review & editing.
